# The influence of growth factors on skin wound healing in rats^☆^

**DOI:** 10.1016/j.bjorl.2015.09.011

**Published:** 2016-01-07

**Authors:** Elen Carolina David João De Masi, Antonio Carlos Ligocki Campos, Flavia David João De Masi, Marco Aurelio Soatti Ratti, Isabela Shin Ike, Roberta David João De Masi

**Affiliations:** a Universidade Federal do Paraná (UFPR), Curitiba, PR, Brazil; b Pontifícia Universidade Católica do Paraná (PUCPR), Curitiba, PR, Brazil; c Universidade da Região de Joinville (Univille), Joinville, SC, Brazil

**Keywords:** Wounds in rats, Growth factor, Healing, Feridas em ratas, Fator de crescimento, Cicatrização

## Abstract

**Introduction:**

Healing is a process that restores the physical integrity of body structures. It is a dynamic, complex, multicellular process that involves the extracellular matrix, cytokines, blood cells, and growth factors. Growth factors are proteins that activate and stimulate cell proliferation through the activation of angiogenesis, mitogenesis, and gene transcription, accelerating the healing process.

**Objective:**

To assess the influence of growth factors on the healing process of wounds made on the backs of female rats compared to the control wound, through macro and microscopy.

**Methods:**

This study used 45 female Wistar rats, in which three wounds were made on the back. The first was the control wound, the second received epithelial growth factor injection, and the third received a combination of factors. Macroscopic and microscopic assessments were performed on the third, seventh, and 15th days of the experiment. For microscopic analysis, hematoxylin–eosin staining was utilized to assess the inflammatory process; vimentin, for assessment of blood vessels and fibroblasts, and Sirius Red for collagen assessment.

**Results:**

In the macroscopic assessment, the use of growth factors resulted in faster healing and decrease of granulation tissue on days seven and 15; (80.31% reduction in the control wound *vs.* 83.24% in the epithelial wound *vs.* 100% in the mixed wound). Utilizing microscopy, at the three stages of the experiment, there were no significant differences between the three wounds; however, when comparing the day of euthanization for each type of wound, there was a favorable outcome for epithelial and mixed wounds (between the third *vs.* 15th day, *p* < 0.001, and in the comparison of the seventh *vs.* 15th day; *p* = 0.002 and *p* = 0.001 for epithelial and mixed wounds, respectively) with a higher number of fibroblasts, angiogenesis, and collagen type I.

**Conclusion:**

The use of growth factors accelerates healing, stimulates greater angiogenic activity, and accelerates fibroplasia and collagen maturation.

## Resumo

**Introdução:**

A cicatrização é um processo de restauração da integridade física das estruturas do corpo. É um processo dinâmico, complexo, multicelular que envolve matriz extracelular, citosinas, células sanguíneas e fatores de crescimento. Os fatores de crescimento são proteínas que estimulam e ativam a proliferação celular mediante a ativação da angiogênese, mitogênese, transcrição genética, acelerando o processo de cicatrização.

**Objetivo:**

Avaliar a influência dos fatores de crescimento no processo cicatricial de feridas realizadas no dorso de ratas em comparação com a ferida, controle através da macro e microscopia.

**Método:**

Foram utilizadas 45 ratas *Wistar*, submetidas à criação de três feridas no dorso. A primeira controle a segunda com injeção de fator de crescimento epitelial e a terceira com fator misto. As avaliações macroscópicas e microscópicas foram realizadas no 3°, no 7° e no 15° dia do experimento. Para análise microscópica, utilizou-se coloração de Hematoxilina-Eosina para avaliar o processo inflamatório; vimentina, para a avaliação dos vasos e fibroblastos, e *Sirius Red*, para avaliar o colágeno.

**Resultados:**

Na avaliação macroscópica, o uso de fatores de crescimento proporcionou cicatrização mais rápida e diminuição do tecido de granulação no 7° e 15° dia; (80,31% de redução na ferida controle *vs*. 83,24% na ferida epitelial *vs*. 100% na ferida mista). Na microscopia, nos três momentos do experimento, não foram encontradas diferenças significativas entre as três feridas; entretanto, quando comparados os dias de morte em relação a cada tipo de ferida, observou-se resultado favorável para as feridas epiteliais e mistas (entre 3°×15° dia apresentou p < 0,001 e na comparação entre 7°×15° dias; p = 0,002 e p = 0,001 para as feridas epiteliais e mistas) com maior número de fibroblasto, angiogênese e colágeno tipo 1.

**Conclusão:**

a utilização de fatores de crescimento acelera a cicatrização, estimula maior atividade angiogênica, acelera a fibroplasia e maturação do colágeno.

## Introduction

Healing is a process that restores the internal and/or external physical integrity of body structures and involves complex interactions between cells and several other factors. It is a dynamic and complex process, consisting of three phases: tissue inflammation, proliferation, and remodeling.^1^ The healing process comprises the extracellular matrix, cytokines, blood cells, and growth factors. Growth factors are proteins that stimulate and activate cell proliferation through activation of angiogenesis, myelogenesis, and gene transcription, among other reactions, which activate and accelerate the healing process.^1^, ^2^

Among the growth factors, the most important ones for wound healing include: epithelial growth factor (EGF), platelet-derived growth factor (PDGF), transforming growth factor (TGF-b), vascular endothelial growth factor (VEGF), fibroblast growth factor (FGF), and insulin growth factor (IGF); the latter stimulates cell proliferation, tissue remodeling, and collagen and elastin increase. VEGF acts on angiogenesis and tissue granulation at the early stage of healing. PDGF is crucial for inflammation, granulation, re-epithelialization, and remodeling in the three stages of wound healing.^3^, ^4^

Due to the pathological and physiological complexity of the healing process, the perfect regeneration of tissues is difficult to achieve.^4^, ^5^ Therefore, the assessment of new treatments is needed, as well as the use of new strategies. The use of growth factors and their combinations have been suggested as promising treatments, because they accelerate the healing process. However, a major obstacle is that the growth factors are degraded by proteinases or removed by exudates before they reach the wound bed.^5^

A great number of growth factors and cytokines are present at the wound site. Their dynamic expression manifests temporal and spatial characteristics in the regulation and changes in the pattern of expression of growth factors that are associated with impaired wound healing. Important alterations in the levels of one factor eventually affect the production of other growth factors and cytokines. Thus, it has been shown that pro-inflammatory cytokines and growth factors are released in serum during the early phase of wound healing, and act as potent stimulators of the expression of several other growth factors. One example is the regulation of FGF7, a growth factor produced by fibroblasts at the wound site. Another example is the regulation of VEGF, a major regulator of angiogenesis, which is produced by keratinocytes and macrophages at the wound site. It was found that pro-inflammatory cytokines can induce VEGF expression in both cell types. These examples highlight the complex interactions that occur during wound healing. Such interactions should be considered when interpreting the results obtained by the overexpression or elimination of a single growth factor at the wound site.^6^

A study performed to compare the effectiveness of platelet-rich plasma (PRP) in the healing of wounds in rabbits compared two groups: one that received chondrocytes + PRP and the other that received only PRP. These components were subcutaneously injected on the dorsal region of the rabbits; as control, only PRP was injected in four rabbits. After two months they underwent magnetic resonance imaging (MRI) assessment, histological analysis, and quantification of glycosaminoglycans. The MRI showed formation of new cartilage, indicating that PRP regenerates the cartilage, and demonstrating the potential use of this method for the reconstruction of cartilage defects.^5^, ^7^

Shi^8^ showed that the conjunctival growth factor activation and scar formation in corneal wounds in rabbits markedly improved the architecture of the corneal stroma and reduced scar formation. However, the authors concluded that there is no measurable *in vivo* impact of the corneal wound scar and it should be considered as a specific target of drug therapy for corneal scar.

Feng et al.^9^ carried out a study in diabetic rats; to do so, they made wounds on the animals’ backs and injected keratinocyte growth factors to assess healing. Two wounds measuring 2 cm in diameter were made on each side of the spinal column; growth factor was injected in one side, whereas saline solution was injected in the other side. The study period lasted four weeks and photographs were taken daily over a period of 28 days. The results showed cell proliferation and a significant healing stimulus in the wounds with growth factor.

The present study used EGF alone, and VEGF growth factor together with IGF and FGF – which we called mixed factors (MF) – to quantify the collagen, elastin, vessels, and cell proliferation in the skin healing in rats, as well as the effectiveness of growth factors in the healing process in relation to the control wound, that did not receive growth factor or saline injection, to mimic natural healing.

The objective of this study was to assess the influence of growth factors on the healing process through macroscopic evolution, and microscopy of the wound healing process on the backs of female rats that had been injected with EGF or VEGF combined with MF, and to compare the results to control wounds that did not receive injection of growth factors.

## Methods

The animals were handled according to the Brazilian College of Animal Experimentation (Colégio Brasileiro de Experimentação Animal – COBEA) criteria and the requirements established in Guide for the Care and Use of Experimental Animals (Canadian Council on Animal Care). The study was approved by the Ethics Committee on Animal Use (Comissão de Ética no Uso de Animais – CEUA), Decree 787/03-BL of June 11, 2003, under Process No. 23075.013736/2012-11. The study was conducted from July 2012 to July 2015.

Forty-five female Wistar rats were used (*Rattus norvegicus albinus, Rodentia mammalia*), aged between 115 and 130 days, weighing 200–253 g. After they were weighed, the animals were randomly divided into three groups of 15 animals. Healing was assessed at different stages in each group. In Group 1, healing was evaluated on the third day; in Group 2, on the seventh day, and in Group 3, on the 15th day.

Three wounds were made: one measuring 1 cm in diameter and two measuring 0.6 cm. The wounds were made in different sizes in order to measure their tension. Due to non-availability of a tensiometer, wound tension was not performed and the study was carried out by evaluating the macroscopic and the microscopic aspects. In each animal, the excision extended from the skin to the muscle layer.

The wounds were named 1–3 (F1, F2, F3). F1 was the control wound (proximal), F2 (central or medial), received an injection of EGF, and F3 (distal) received an injection of VEGF combined with FGF and IGF (which we called mixed factor MF).

**Figure 1:**
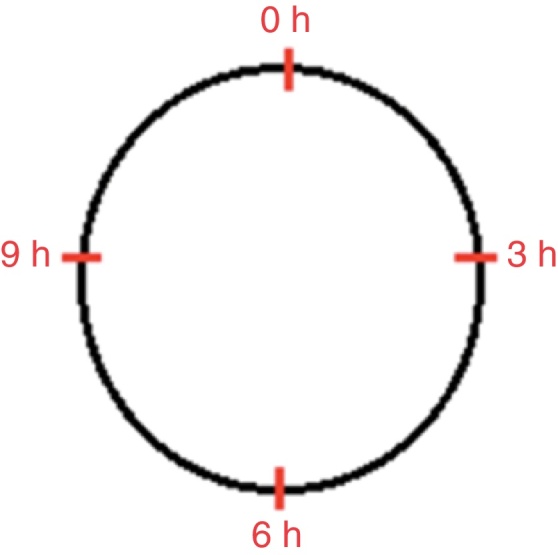
Schematic drawing showing the location of growth factor injection in the epithelial and mixed wounds in the four quadrants: three, six, nine, and 12 o’clock.

**Figure 2:**
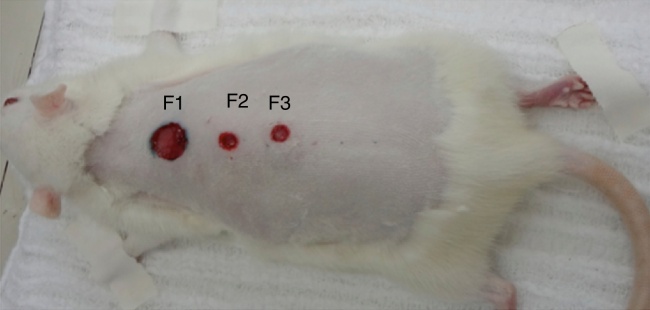
Denomination of the wounds: wound 1 – control (1.0 cm in diameter); wound 2 – epithelial (0.6 cm in diameter), and wound 3 – mixed (0.6 cm in diameter).

The growth factors were injected only on the day the wounds were made. Four injections of 0.5 mL were given for each wound at four points: three, six, nine, and 12 o’clock into the dermis and subcutaneous tissue (Figure 1, Figure 2). The wounds were allowed to heal spontaneously.

The wounds were circular, in order to be able to measure their diameter through photographs, using a common millimeter ruler. The wound diameter results were placed in a graph with the percentages of the respective measurements obtained.

After the euthanization of the animals on the third, seventh, and 15th days of the experiment, the skin-aponeurotic flaps containing the wounds were removed. Subsequently, the material was sampled and submitted to an automated histological process and embedded in paraffin. Histological slides containing the sections were stained by hematoxylin and eosin (HE) and Sirius Red. Additional sections were prepared for the immunohistochemistry analysis, using the antivimentin antibody.

Visualizing the sections stained with hematoxylin and eosin, the number of polymorphonuclear neutrophils, chronic inflammatory cells (lymphocytes and plasma cells), newly-formed capillaries, fibroblast proliferation, and amount of deposited fibers were assessed. Epithelial regeneration was considered as partial or complete.

Sections stained in Sirius Red were used to assess the presence and type of collagen (mature or immature) through polarized light microscopy, quantifying both types of collagen as percentages (immature collagen = green; mature collagen = red).

Histological sections, submitted to the immunohistochemical study and marked with antivimentin antibody, were used to identify fibroblasts and newly formed capillaries.

After assessing the macroscopic and microscopic aspects of F1, F2, and F3, they were compared to the HE staining for all study variables (neutrophils, lymphocytes, macrophages, fibroblasts, vessels) and a separate analysis was performed for each moment of assessment (three, seven, and 15 days) and submitted to statistical analysis, considering the non-parametric Friedman test. Times of euthanization were compared using the nonparametric Kruskal–Wallis test. *p*-Values <0.05 were considered statistically significant. Data were analyzed using Statistica v. 8.0 software.

## Results

**Figure 3:**
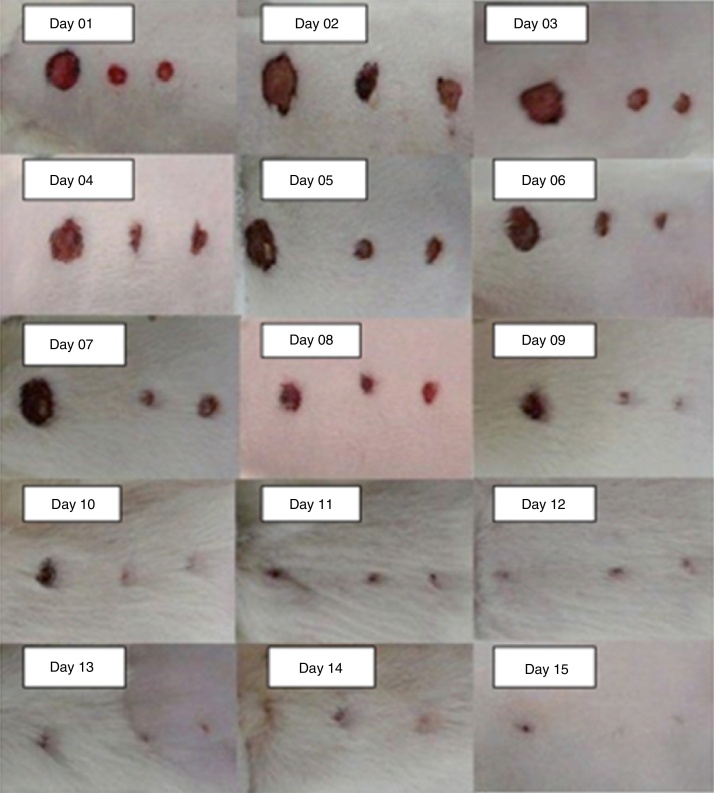
Pictures of wounds from the first to the 15th day of the experiment, taken of different animals.

The macroscopic wound assessment was performed daily and recorded in photographs. The F1, F2, and F3 of all the rats were measured using a ruler graduated in millimeters positioned at the level of the lesion, and the means of all wounds were taken on each day of the experiment (Fig. 3).

On the third day of the experiment, all wounds were open; in F1, there was little wound contraction and granulation tissue; in F2 and F3, there was a slight improvement in relation to F1.

On the seventh day of the experiment, the wounds were still open with a slight contraction of all wounds, but F1 had granulation tissue, showing delayed wound healing.

**Figure 4:**
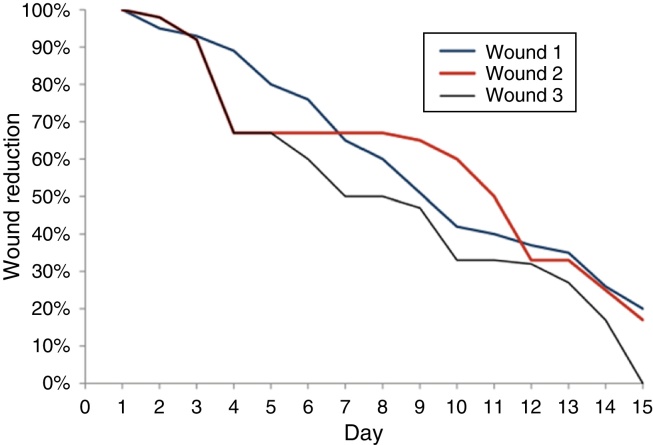
Chart comparing of the healing of wounds 1, 2, and 3 by measuring the wounds 1, 2, and 3 in percentages, throughout the 15 days of the experiment.

On the 15th day of the experiment, the wound scars were as follows: in F1 there was a reduction of 80% ± 0.311% of the wound diameter; in F2, the reduction was 83% ± 0.201%, and in F3 it was 100% (Fig. 4).

When comparing the three types of wound on each day of the animals’ euthanization, there were no significant differences in the number of neutrophils (Table 1). However, when evaluating the progress of each wound on the three days of the animals’ euthanization, a significant difference was found for F1, which showed the highest number of neutrophils. For this wound, there were differences between the third and seventh days (*p* = 0.029) and between the seventh and 15th days (*p* = 0.007). Between the third and 15th days there was no significant difference (*p* = 0.557). For F2 and F3, the results were lower, with a tendency to significance among the three days of assessment (*p* = 0.058 and *p* = 0.076, respectively).

**Table 1:** Neutrophil assessment with hematoxylin and eosin on the third, seventh, and 15th days in each wound; assessment between the groups.

Euthanization day	Wound	*n*	Mean ± SD	*p*^a^
Third day	Control	15	24.2 ± 4.5	0.482
	Epithelial wound	15	21.7 ± 5.7	
	Mixed wound	15	22.9 ± 5.2	
Seventh day	Control	15	31.6 ± 8.4	0.155
	Epithelial wound	15	24.8 ± 5.5	
	Mixed wound	15	28.5 ± 9.6	
15th day	Control	15	23.1 ± 9.6	0.856
	Epithelial wound	15	19.9 ± 4.4	
	Mixed wound	15	20.7 ± 6.2	

a Nonparametric Friedman test, *p* < 0.05; *n*, number of animals; SD, standard deviation; *p*, statistical test results.

Regarding the lymphocytes, when comparing F1, F2, and F3 in each of the euthanization moments, it was observed that there was no significant difference among them (*p* = 0.270). As for the seventh day, there was also no significant difference between the three wounds (*p* = 0.759). The same was observed for the 15th day (*p* = 0.451).

**Figure 5:**
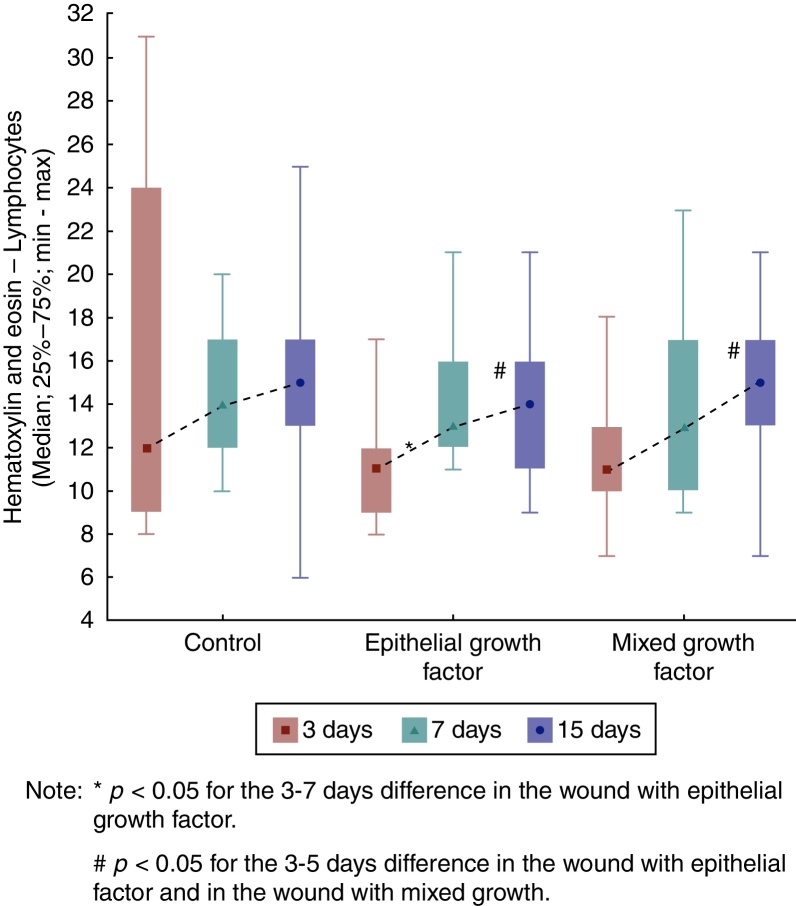
Hematoxylin and eosin evolution – lymphocytes in each wound, assessment.

In F1, the results were similar regarding lymphocytes in the three days of comparison (*p* = 0.774). As for F2, an increase in the number of lymphocytes was observed between the third and seventh days (*p* = 0.007) and between the third and the 15th days (*p* = 0.007). When comparing the assessment days for F3, an increased number of lymphocytes were found only when comparing the third and 15th days (Fig. 5).

Regarding macrophages, the results were similar among the three wounds for both the third and the seventh days, (*p* = 0.247 and *p* = 0.344, respectively). As for the 15th day, the results showed a significant difference among the three wounds, with a lower number of macrophages in F2 and F3 (*p* = 0.005). In addition, for the 15th day, when comparing the wounds two by two, a difference was observed between F1 and F2 (*p* = 0.001), with fewer macrophages in F2; and between F1 and F3, there were fewer macrophages in F3 (*p* = 0.004). F2 and F3 showed no significant difference (*p* = 0.660; Table 2).

**Table 2:** Macrophage count per field and comparison of wounds 1, 2, and 3 at each moment of euthanization.

Euthanization day	Wound	*n*	Mean ± SD	*p*^a^ value (F1 × F2 × F3)
Third day	Control	15	28.2 ± 10.5	0.247
	Epithelial wound	15	26.9 ± 10.6	
	Mixed wound	15	25.9 ± 10.1	
Seventh day	Control	15	45.5 ± 9.9	0.344
	Epithelial wound	15	44.8 ± 13.5	
	Mixed wound	15	48.1 ± 10.7	
15th day	Control	15	33.9 ± 5.5	0.005
	Epithelial wound	15	25.8 ± 10.1	
	Mixed wound	15	27.3 ± 8.1	

a Nonparametric Friedman test, *p* < 0.05.

**Figure 6:**
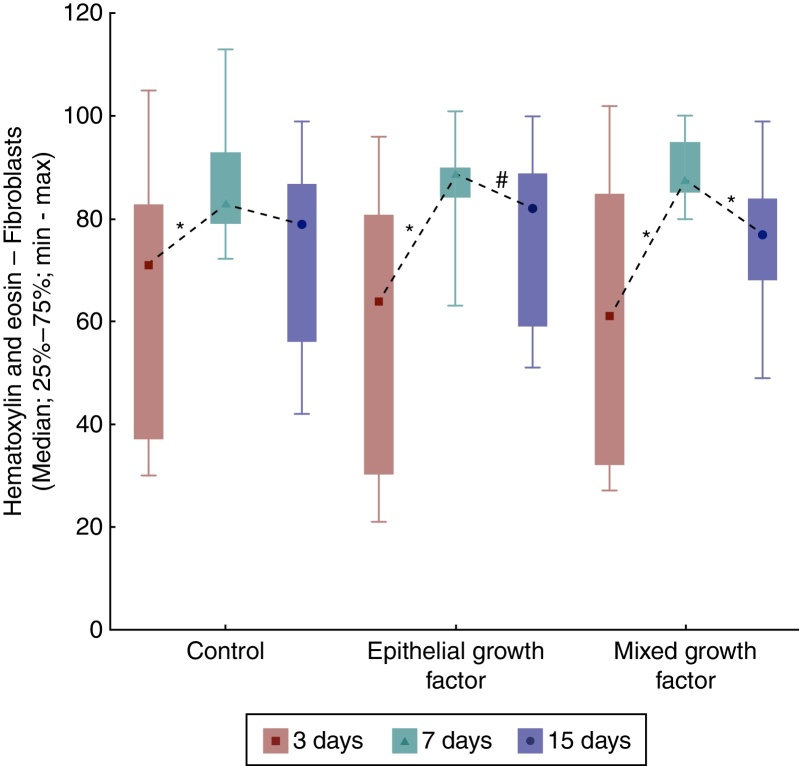
Hematoxylin and eosin evolution – fibroblasts in each wound, between the times of euthanization, assessment between groups.

When comparing the days of euthanization regarding fibroblasts, significant differences were obtained among them, with the highest number of fibroblasts in F2 and F3 (*p* = 0.018 for F1; *p* = 0.003 for F2, and *p* < 0.001 for F3). When comparing the days of euthanization two by two, there were differences between the third and seventh days (*p* = 0.004 for F1 and *p* < 0.001 for F2 and F3, which showed higher number of fibroblasts on day seven). When comparing between the third and 15th days, a significant difference was verified only for F2 (*p* = 0.044) and in the comparison between the seventh and 15th days, only for F3 (*p* = 0.003; Fig. 6).

**Figure 7:**
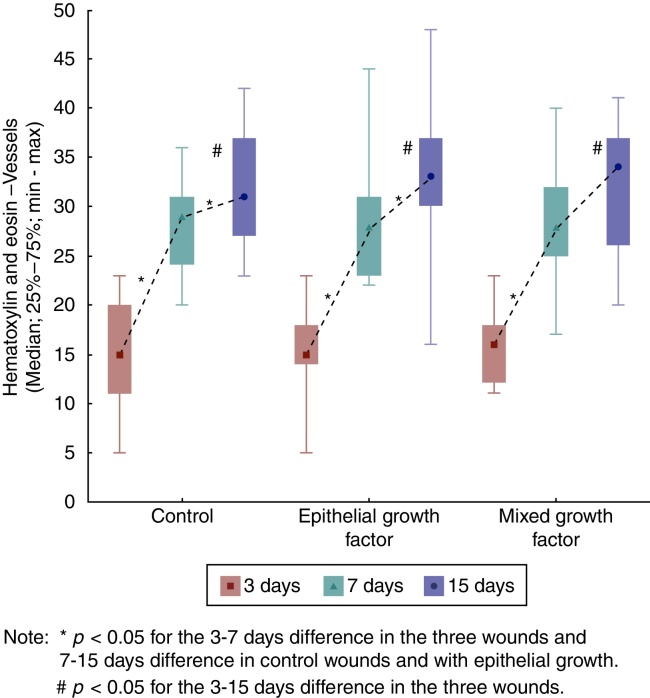
Hematoxylin and eosin evolution – vessels in each wound, evaluation between the times of euthanization, intra-group assessment.

When assessing the evolution of each wound in the three days of euthanization, significant differences were found regarding the increase in the number of vessels (*p* < 0.001). When comparing the days of euthanization in pairs, significant differences were observed between the third and seventh days (*p* < 0.001), and there were also significant differences between the third and 15th days (*p* < 0.001). Between the seventh and 15th days, there was no significant difference for F1 and F2 (*p* = 0.044 and *p* = 0.035, respectively) (Fig. 7).

**Figure 8:**
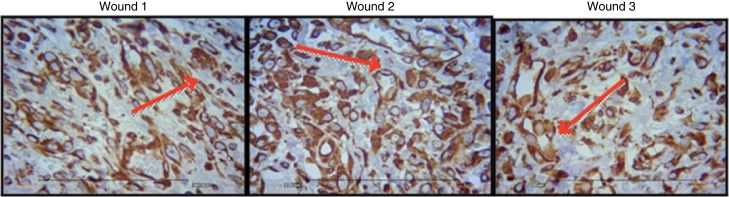
Photomicrograph of vimentin – in which macrophages, fibroblasts, and vessels are evaluated, on the third day of evolution, in the wounds 1, 2, and 3 (VIM × 200).

**Figure 9:**
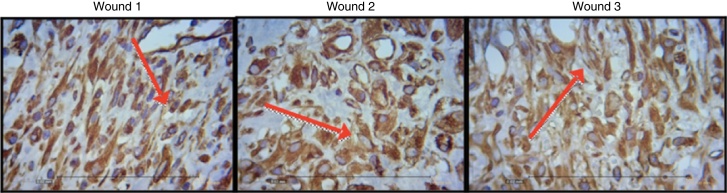
Photomicrograph of vimentin – where macrophages, fibroblasts, and vessels are evaluated, on the seventh day of evolution, in wounds 1, 2 and 3 (VIM × 200).

**Figure 10:**
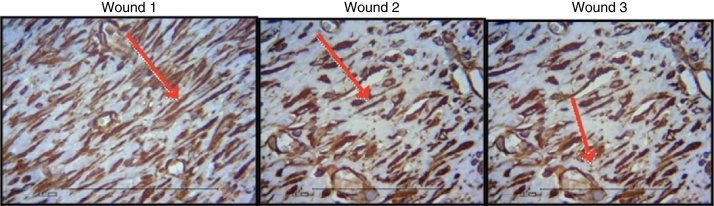
Photomicrograph of vimentin – where macrophages, fibroblasts, and vessels are evaluated, on the 15th day of evolution, in wounds 1, 2, and 3 (VIM × 200).

Figure 8, Figure 9, Figure 10 show the histological details of the immunohistochemical assessment of scars in all three assessed periods of time.

**Figure 11:**
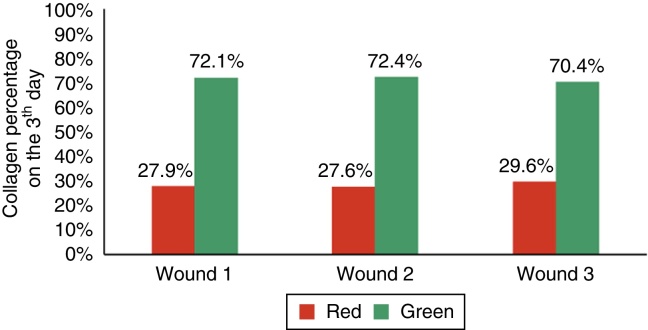
Demonstration of the percentage of type I and type III collagen on the third day of the experiment in the three wounds.

**Figure 12:**
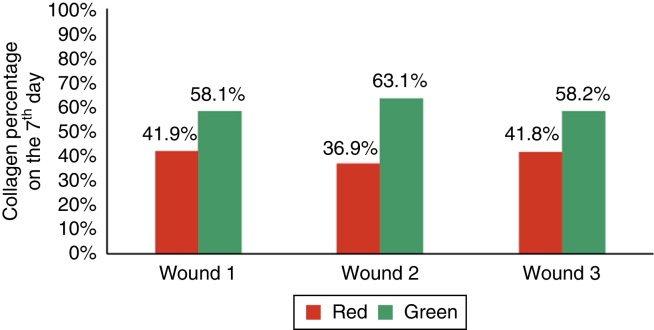
Demonstration of the percentage of type I and type III collagen on the seventh day of the experiment in the three wounds.

**Figure 13:**
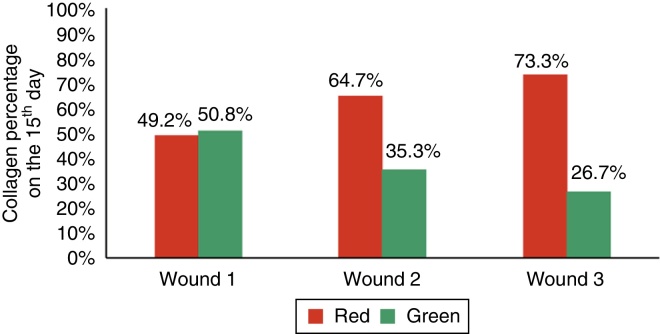
Demonstration of the percentage of type I and type III collagen on the 15th day of the experiment in the three wounds.

When assessing type I collagen on the days of euthanization, there was no statistical significance for F1 (*p* = 0.240), whereas there was a greater amount of type I collagen for F2 and F3 (*p* ≤ 0.001), demonstrating a decrease in the amount of inflammatory infiltrate, increased angiogenesis and fibroplasia, with a faster and more organized healing (Figure 11, Figure 12, Figure 13).

Regarding collagen type III, significant differences were found in F2 and F3, with a lower amount of type III collagen (*p* ≤ 0.001).

## Discussion

In recent decades greater understanding of the concepts related to healing have mobilized the industry to develop and commercialize more specific products that are effective and adequate for each type of wound with respect to the cost/benefit ratio. Currently, researchers are evaluating changes in molecular biology concerning the synthesis of substances involved in the healing phenomena.^10^ With so many resources to promote healing, in this study it was decided to evaluate growth factor injections in wound healing, as they positively affect the healing process.

Wound healing is a complex series of reactions and interactions of inflammatory mediators and cell growth interactions.^10^, ^11^, ^12^, ^13^, ^14^ Many intrinsic and extrinsic factors affect wound healing, and there is a great variety of commercial options that aim to counteract negative interference or stimulate the healing process.^14^ The interrelationship between nutrition and the wound healing process acts together with the immune system and the immunomodulatory function.^15^ The present study analyzed the healing process evaluating macroscopic and microscopic aspects, as well as the influence of growth factors on the healing process. In the beginning of the healing process, there is a migration of neutrophils stimulated by platelets, and after that the macrophages, which contribute to the angiogenesis and fibroplasia. This occurred in the present study, in which the wounds with EGF and MF infiltration exhibited an increased number of neutrophils, with *p* = 0.058 and *p* = 0.076; for the macrophages, significant differences were found in the three wounds (*p* < 0.01); but when comparing the wounds two by two, there was significant difference for the F2 and F3 (*p* < 0.001). In the HE evaluation, regarding the vessels, there were significant differences between the three wounds (*p* < 0.001). The immunohistochemical analysis also showed increased fibroblasts, macrophages, and vessels for wounds F2 and F3 (*p* < 0.001). For type I collagen, F2 and F3 showed greater deposition of this type of collagen (*p* = 0.001 and p < 0.001), demonstrating greater inflammatory response and increased angiogenesis, with faster healing. The type I collagen percentages on the 15th day of the experiment were: for F1 (49.2%), for F2 (64.7%), and for F3 (73.3%). This demonstrates the improvement in wound healing where there was injection of growth factors in relation to the control wound.

With regard to growth factors derived from platelets, van den Dolder et al. carried out a study in rat bone matrix and performed an *in vitro* culture, a sample of bone matrix covered with skin and PRP and another sample with only bone matrix with skin; they concluded that PRP stimulates the matrix and bone differentiation, as a positive correlation was found between wound healing and growth factor.^16^ The present study analyzed the use of VEGF, EGF, FGF, and IGF to accelerate wound healing; favorable results were obtained when they were compared with the control wound, without the presence of growth factors. Wound area was macroscopically analyzed and showed the following results: F1 showed an area of 0.031 cm^2^ at the end of the experiment and the F2 and F3 areas were, respectively, 0.008 cm^2^ and 0, demonstrating a more effective healing.

The percentage of collagen in the present study, observed through the microscopy using HE, was higher in wounds with growth factor, in addition to a lower inflammatory response when compared with the control wound (*p* < 0.05 = 0.016). Similar results are found in the literature. Xie et al. used a rat model and compared healing in wounds treated with growth factors and a control wound. The wounds were monitored daily and measured with a caliper on days one, seven, 14, and 28, when euthanasia and excisional biopsy were performed. The microscopic analysis was performed with HE and collagen was measured in percentages.^3^ The methodology was similar to that used in this study for the creation of the wounds, measurements, biopsy, and microscopy. The results were also similar, as they showed a higher percentage of collagen in wounds with growth factor.

Another article studied 0.8 cm wounds on the backs of diabetic rats, performed by punch and with topically-applied endothelial progenitor cells immediately after surgery, with a separate wound used as control. Measurements were performed on days five, ten, and 14 after the treatment.^17^ As in this study, the wounds were also resected and the vascularization histological analysis was performed, with a *p*-value <0.001 in the intragroup comparison, between the third and seventh days, and third and 15th days among the three wounds; between the seventh and 15th days there was a significant difference for F1 and F2; *p* = 0.044 and *p* = 0.035, respectively. The study by Asai et al. showed that topical use of growth factor promoted healing improvement, as demonstrated in the present study.^17^

In another study, the authors compared a wound healing model in Wistar rats through the expression of growth factors – Group 1: healthy without wound; Group 2: excisional wound; Group 3: transcutaneous electrical nerve stimulation (TENS); Group 4: topical saline solution; Group 5: povidone iodine; Group 6: lavender oil. The experiment lasted five days and the growth factors were measured by immunoassay and immunohistochemistry analysis. The levels of PDGF and EGF growth factors were significantly higher in the TENS group compared to the other groups and the control group (*p* < 0.05).^4^ This study showed the different treatments for wound healing, distinct from the present study in which only the effect of growth factors on wound healing was assessed; there was no external stimulus, only growth factors were used.

It is noteworthy that although the present study obtained favorable results with the use of EGF and MF (*p* < 0.05) on the three days of assessment and a higher percentage of mature collagen for F2 and F3, with 64.7% and 73.3%; respectively, when compared with F1 with only 49.2%, there was a study that evaluated the effects of PRP in horse wounds with negative results. Three wounds were created in the metacarpal region of six mares of different breeds, totaling 36 wounds; of these 36 wounds, 18 were treated with PRP and bandage and 18 were control wounds, treated only with bandage without medication. An anatomopathological study was performed one, two, and three weeks after the wound was made. A large amount of granulation tissue and high concentration of transforming growth factor B1 were observed in the wounds treated with PRP, when compared with wounds that were not treated with PRP. Histological, biochemical data, and gene expression did not significantly differ between wounds treated with PRP and the untreated. The authors concluded that the topical application of autologous PRP does not accelerate or improve healing of wounds with granulation in horse limbs. This kind of treatment can improve wounds with extensive tissue loss or alternatively, chronic wounds that would benefit mediators to accelerate the healing process.^18^

The care and objectives of the study by Pradeep consisted in comparing the clinical effectiveness of two regenerative techniques in the treatment of bone defects in humans. PRP and PRP associated with inorganic matrix derived from bovine tissue and peptide-15 (ABM/P-15) were used. Analyzing the clinical and radiological parameters in 14 patients, they created bone defects and injected PRP, and in another group, PRP associated with AMB/P-15. All analyzed clinical parameters (plaque index, groove bleeding index, level of insertion, gingival margin level, probing depth) showed a statistically significant difference (*p* < 0.001) for the graft group associated with PRP. The computerized tomography (CT) scan showed that bone growth was significantly better when PRP + AMB/P-15 were combined (p < 0.001). It was concluded that the combination of PRP and AMB/P-15 was more effective than PRP alone in treating intraosseous defects.^19^ Similar results were obtained in the present study, as the wounds with MF showed slightly better healing in relation to wound with EGF, which leads to the conclusion that a combination of growth factors results in better healing.

## Conclusion

The macroscopic results revealed a reduction of the open wound area, and the microscopic findings support the conclusion that the use of growth factors injected into the borders of skin wounds in rats, both epithelial growth and a combination of vascular endothelial growth factor, fibroblast growth factor and insulin growth factor, accelerate the healing process compared to the control wound, as they foster greater angiogenic activity, and accelerated fibroplasia and the deposition of type I collagen, in addition to accelerating the maturation of healing.

## Conflicts of interest

The authors declare no conflicts of interest.
